# Book Reviews

**Published:** 1916-09

**Authors:** 


### BOOK REVIEWS

Books mentioned may be inspected at and ordered through this office. So far as possible, books received in any month will be reviewed in the issue of the second month following.

---

**Parke, Davis & Co.'s Chinese Catalogue.**

This was prepared in the U. S. sent to China and translated into Chinese, retranslated into English and revised. It is copiously illustrated, with cuts of original packages, scenes from the Detroit laboratories and some local views of Tashmoo. A deer's head has been adopted as the Chinese trade-mark. Excepting the headings and the firm's name and address, we are unable to vouch for the reading matter but it is certainly decorative. The catalogue is a linguistic curiosity but even more noteworthy is the economic unity of the world which has rendered such a translation necessary.

---

**Examination of the Urine and Other Clinical Side-Room Methods.** Andrew Fergus Hewat, M. B., Ch. B., M. R. C. P., Edinburgh. 5th edition. Printed in England. Issued by Paul B. Hoeber, 67 and 69 E. 59, N. Y. 212 pages, vest-pocket size, $1.

This is a very convenient little manual, surprisingly complete for urine, blood, sputum, pus, gastric contents and faeces.

---

**Modern Medicine** and Some Modern Remedies, Thomas Bodley Scott, Bournemouth, Eng., with preface by Sir Lauder Brunton. Paul B. Hoeber, 67 and 69 E. 59, N. Y. 159 pages, $1.50.

This consists of essays on Disorders of the Heart, Arteriosclerosis, Therapeutic Speculations and Doubts and Chronic Bronchitis and Bronchial Asthma. Under therapeutic speculations, mention is made of hormones, endocrinic organs, glandular extracts, etc. The work contains many practical suggestions and even the speculative portion is of value as calling attention to the development of therapeutics away from the old line of inorganic drugs. We trust that it will arouse sufficient interest to lead to the issue of a much enlarged edition, which would probably require a corps of writers, to cover the various phases of modern therapeutics completely.

---

**United States Life Tables**: 1910 Bureau of the Census; Supervision of Prof. James W. Gliver, University of Michigan.

These are based on statistics of the registration states and show the average number surviving at any age, of an initial population of the same age; the average deaths each year, expectation of life, etc. These statistics apply to the general population and groups, by sex, race, etc. Earlier life tables have been based either on rather remote statistics, or have been modified by the factor of financial safety and profit on the one hand, or the better showing of selected risks on the other hand, when actuaries' tables have been used to solve problems in vital statistics. This work, therefore, is of the utmost value as furnishing a dependable and very nearly accurate basis for study of various problems in longevity, etc.

**Ultra-Violet Light,** by means of the Alpine Sun Lamp, Treatment and Indications. Hugo Bach, M. D., Bad Elster, Saxony. Authorized translation. Paul B. Hoeber, N. Y. 114 pages, $1.00.

The quartz lamp is fully described, the advantages over sun-light being solely in its availability at all times and the concentration of the ultra-violet rays which are largely absorbed by the atmosphere, especially the lower strata under which most of the inhabitants of the world reside. As a by-product, ozone is generated. While a large number of diseases are more or less amenable to photo-therapy, the author is conservative in his claims and emphasizes the necessity of individualizing rather than generalizing.

---

**Diagnosis and Treatment of Heart Disease.** Practical points for students and practitioners. E. M. Brockbank, M. D., F. R. C. P., Manchester, Eng. Paul B. Hoeber, 67-69 E. 59, N. Y. 120 pages, illustrated, $1.25.

This book is exactly what it purports to be. The modern elaborate instrumental methods of studying the heart and alluded to only for illustrative purposes while the clinical methods of investigation that must remain the basis of practice are thoroughly treated. Treatment is briefly but comprehensively discussed under the headings of special forms of heart disease and diet, posture, change of occupation; diuretics and other remotely acting drugs are included.

---

**Annual Report of the Dept. of Health, Buffalo.** Year ending Dec. 31, 1915.

Dr. Fronczak and his staff are to be congratulated on the excellent way in which the manifold services of this department are conducted. This department costs the city about $200,000 a year or about 40 cents per capita. Almost exactly 3/4 of the expense is for salaries. This means, allowing for reasonable salaries and an efficient corps with no padding of the pay-roll, a high degree of economy. In the past 33 years—the average generation—the mortality per thousand has declined from 24.06 to 14.83, quite steadily although an apparent minimum of 12.25 was reached in 1898 on an estimated population undoubtedly excessive. Tuberculosis remains the cause of about 1/10 of all deaths; typhoid causes about 1/6 of the deaths that would have been expected from the growth of the city under the same sanitary conditions as a generation ago. Diphtheria seems to have been reduced to about a quarter of its former mortality but the marked fluctuations in other common infections prevents any conclusion based on an average of several years, save that the epidemics recurring every few years seem to have been considerably reduced. Almost a third of all deaths occur in institutions—which suggests that we are developing a dependent class of considerable magnitude, also that the death rate is being swelled by desperate cases from without. In this connection 923 of the dead were buried out of town while 429 bodies were brought to Buffalo for burial. The bacteriologic reports show a large development of useful diagnostic procedurês. Analyses of tap water varied from 1,440 to 5 per c.c. As an approximate general average, the Dept. of Health consumes about 2% of the total tax and, as stated, it costs about 40 cents per capita per annum. Every physician and every taxpayer should study this report thoroughly and see if he is getting his money's worth. We are, though it happens that we are paying a tax entirely disproportionate to our income.

---

**Text Book of Practical Gynecology.** D. Tod Gilliam, M. D., and Earl M. Gilliam, M. D., Columbus. F. A. Davis Co., Philadelphia. 5th edition, 681 pages, 352 engravings, colored frontispiece and 13 full page plates. $5.00.

This work, already favorably known in its earlier editions, views the subject broadly. General considerations, as of examinations, technic, etc., precede the discussion of diseases in detail. Nearly a quarter of the work is devoted to abdominal and pelvic lesions not strictly gynecologic, to those of the bladder, urethra and ureters, kidneys and rectum. These conditions are practically included in the work of the gynecologist. An index of regional symptoms is presented. The work is well balanced between so-called medical and operative treatment; that is to say, the author does not neglect non-operative procedures, yet advises and describes them for the majority of lesions, in accordance with the best opinion of the times.

---

**Practical Massage and Corrective Exercises.** Hartvig Nissen, Boston. F. A. Davis Co., Philadelphia. 211 pages, 68 original illustrations, $1.50.

This book is a revised and enlarged edition of the author's "Practical Massage in Twenty Lessons." It discusses the general principles of mechano-therapy and gives detailed exercises and methods of massage for various conditions to which such treatment is appropriate.

**Venesection.** Walton Forest Dutton, M. D., Tulsa, Ok. F. A. Davis Co., Philadelphia. 220 pages, illustrated. $2.50.

This includes a paper on the history of blood letting, by Dr. Fielding H. Garrison, and describes the general principles and technic both of venesection and of the blood and blood pressure and analogous methods of diagnosis upon which the indications for blood letting rest. It then discusses in detail the surprisingly numerous conditions in which blood letting is, even at present, to be considered as a potential means of relief.

---

**Cerebellar Abscess.** Etiology, Pathology, Diagnosis and Treatment, including Anatomy and Physiology of the Cerebellum. Isidore Friesner, M. D. and Alfred Braun, M. D., N. Y. Paul B. Hoeber, N. Y. 186 pages, 10 full-page plates and 16 text illustrations. $2.50.

This is one of a considerable series of medical monographs published by Mr. Hoeber, who has made a most careful selection of authors and subjects, with the idea of placing before the profession thoroughly modern critical studies of various subjects on which it is difficult to secure accurate information. At first thought, it might seem that this work would appeal only to neurologists and brain surgeons. But, we recall a visit to a foreign hospital with a distinguished clinician who told of a case that had baffled himself and everyone else, even as to its proper classification, and which was finally cleared up by the chance visit of a man who, apparently without any scientific study of the case, made the diagnosis of cerebellar abscess, although the first impression of those who had observed the case for some time was that this was merely a random guess. We likewise recall a patient in our interne experience, twice treated (?) in a hospital, without any diagnosis at all and purely expectantly, who died suddenly and whose brain showed several tumors, including one of the cerebellum. Cerebellar lesions are undoubtedly rare but they cannot be neglected on that account. The symtomatology is vague. The detection of such cases and the preservation of life depends on a well diffused knowledge of their nature and manifestations. To a larger degree than the majority of rare and puzzling cases, they are liable not to suggest their gravity, not even to suggest the proper special consideration which may unravel their nature. So far as is humanly possible at present, this book places before the profession the means of proper diagnosis and treatment.

**A Text-Book of Pathology.** By William G. MacCallum, M. D., Professor of Pathology in the College of Physicians and Surgeons, Columbia University, New York City. Octavo volume of 1085 pages with 575 original illustrations. Philadelphia and London: W. B. Saunders Company, 1916. Cloth, $7.50 net.

In authorship, as well as in illustration and other phases of the publisher's art, this work comes immediately into the class of comprehensive special text-books of high order. The literary style is brightened by the occasional use of comparisons with non-technical experience. Certain general topics, as atrophy, hypertrophy and degenerations are elucidated by still more fundamental discussions of metabolism, as of carbohydrates, fats, calcium, iron, pigments, etc. The diseases of the ductless glands are especially well illustrated. Destructive and reparative processes are grouped so as to bring out the relations of diseases ordinarily thought of as distinct, for example certain types of nephritis, hepatic cirrhosis, endocarditis, arteriosclerosis which last is sharply divided into arteriosclerosis proper and syphilitic and other secondary forms. Thus, in many ways, original and comprehensive points of view are suggested.

---

**The Practical Medicine Series,** edited by Charles L. Mix, A. M., M. D. The Year Book Publishers, 327 S. La Salle St., Chicago, 10 volumes annually, $10.

Vol. 3, Eye, Ear, Nose and Throat, edited respectively by Casey A. Wood, C. M., M. D., D. C. L.; Albert H. Andrews, M. D. and George E. Schambaugh, M. D. 376 pages, illustrated, $1.50.

Vol. 2, General Surgery, edited by John B. Murphy, A. M., M. D., LL.D., F. R. C. S., 620 pages illustrated.

This comprehensive review of current medical literature is eminently satisfactory. The volume on surgery is, we believe, the largest yet issued. It exceeds many text books that aim to cover the entire field of surgery in condensed fashion. The considerable amount of new experience in military surgery, especially in regard to infections and the application of the X-ray, extensive work in abdominal and central nervous surgery account for the unprecedented increase in original matter worthy of review.

---

**Thirty-Seventh Archaeological Report,** 1915, being part of the Appendix to the Report of the Minister of Education, Ontario.

**Twenty-Ninth Annual Report of the Bureau of American Archaeology,** 1907-8, Ethnography of the Tewa Indians, John Peabody Harrington.

**Thirtieth Annual Report,** same, 1908-9, Ethnobotany of Zuni Indians, Matilda Coxe Stevenson; Inquiry into the Animism and Folk Lore of the Guiana Indians, Walter E. Roth.

An article by W. R. Harris, D. D., LL.D., on the Practice of Medicine and Surgery by the Canadian Tribes in Champlain's Time, in the first publication; the references to medicinal and food plants used by the Zunis and the customs and beliefs of the aborigines regarding obstetrics, and allied topics, are of considerable medical interest.

